# Supplementary figures and images for: Renin angiotensin system genes are biomarkers for personalized treatment of acute myeloid leukemia with Doxorubicin as well as etoposide
Source: PLoS One. 2020 Nov 25;15(11):e0242497. doi: 10.1371/journal.pone.0242497 (PMC7688131; doi:10.1371/journal.pone.0242497)

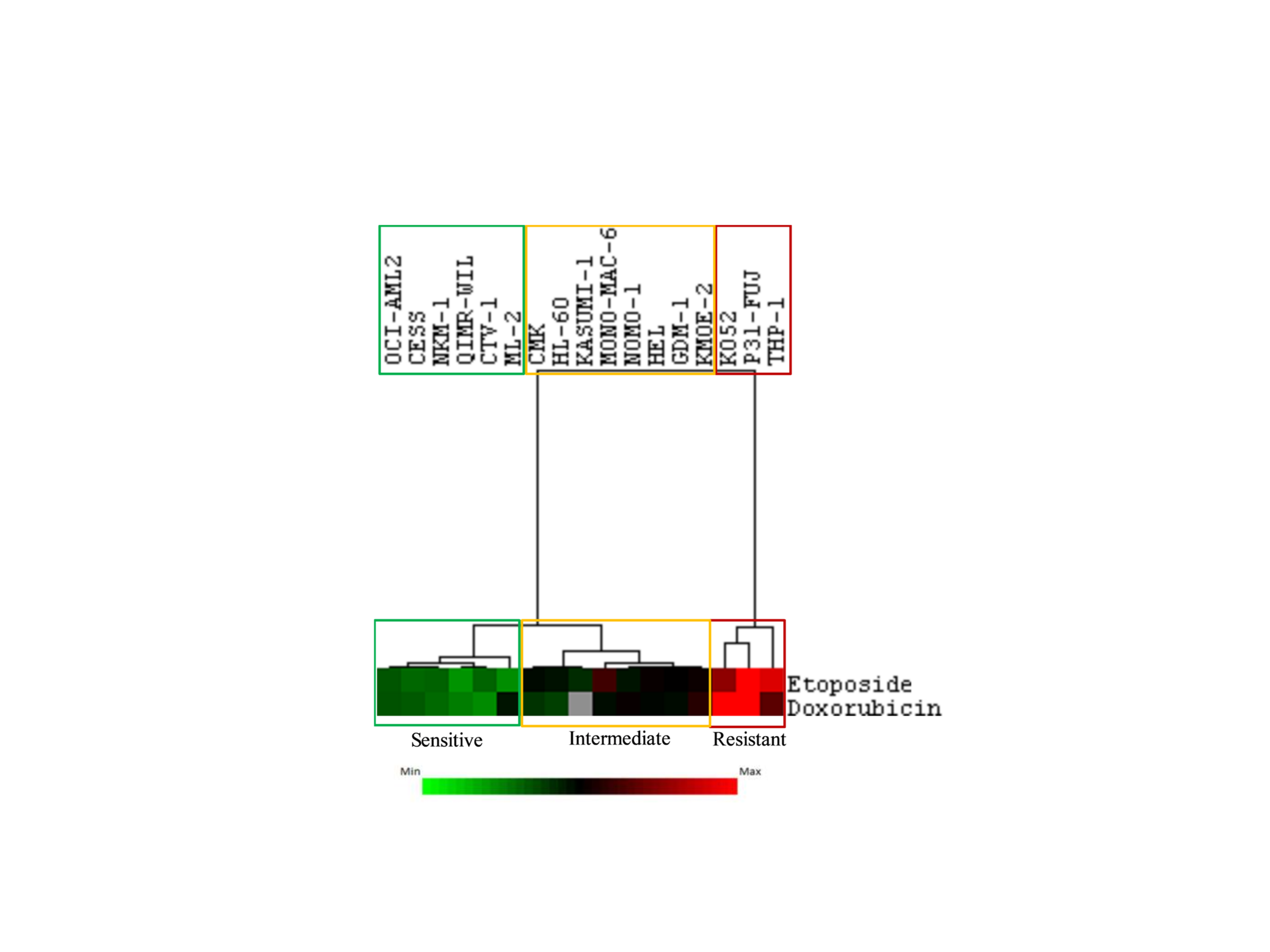

Supplement: S1 Fig — The analysis reveals sensitive (six cell lines-green), intermediate (eight cell lines-orange) and resistant (three cell lines-red) subgroups for the 17 AML cell lines. Sensitivity to Doxorubicin and Etoposide is highly concordant in three subgroups. Green indicate low expression, orange indicate intermediate expression and red indicates high expression. (TIF) [file pone.0242497.s001.tif]

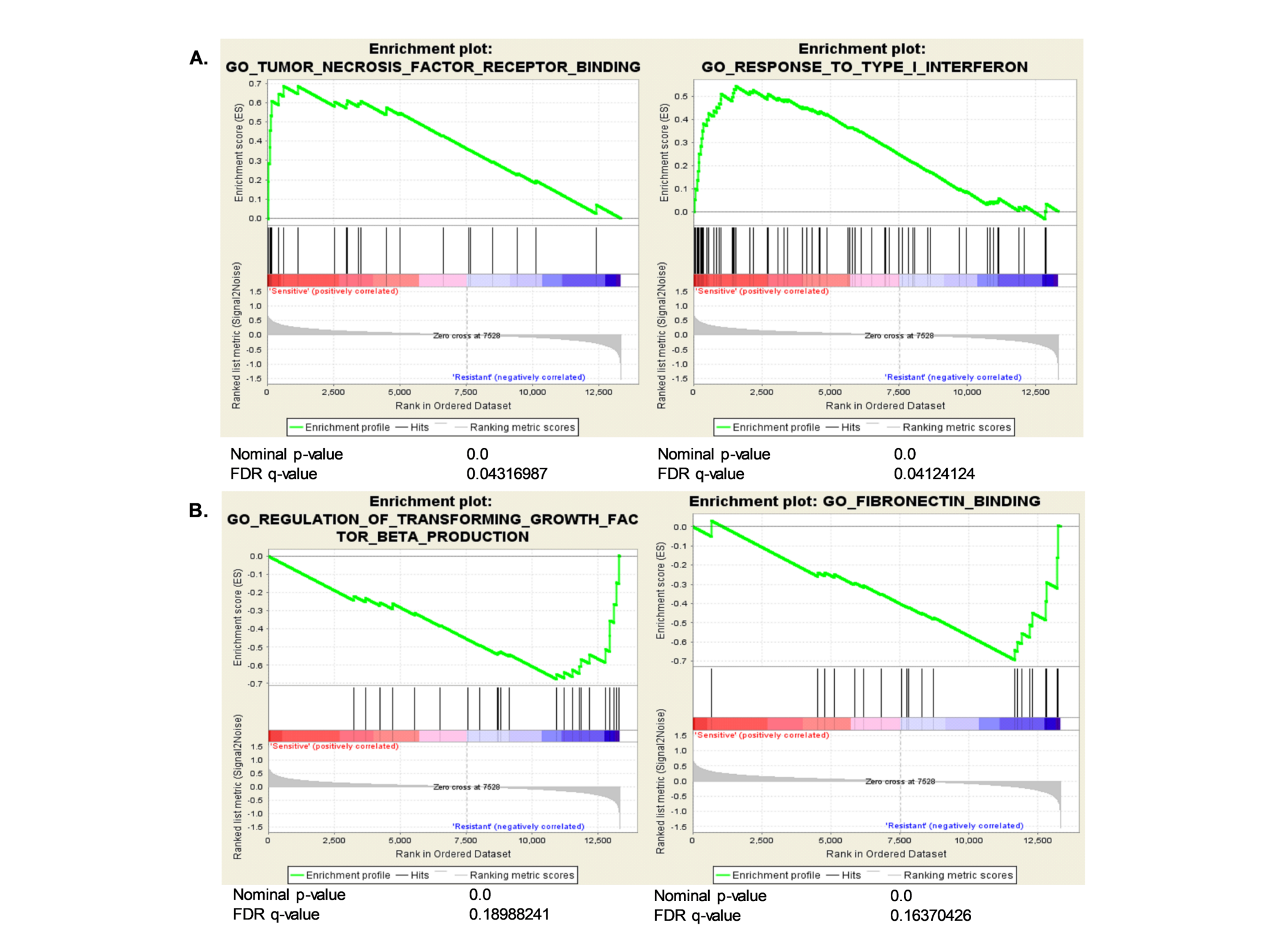

Supplement: S2 Fig — (A) Plots showing gene sets enriched in sensitive cells, including genes interacting with TNF-receptor and genes affected in response to type I IFN stimulus. (B) Plots showing gene sets enriched in resistant cell lines, including genes having role in regulation of TGF-B production and genes interacting selectively and non-covalently with Fibronectin. (TIF) [file pone.0242497.s002.tif]

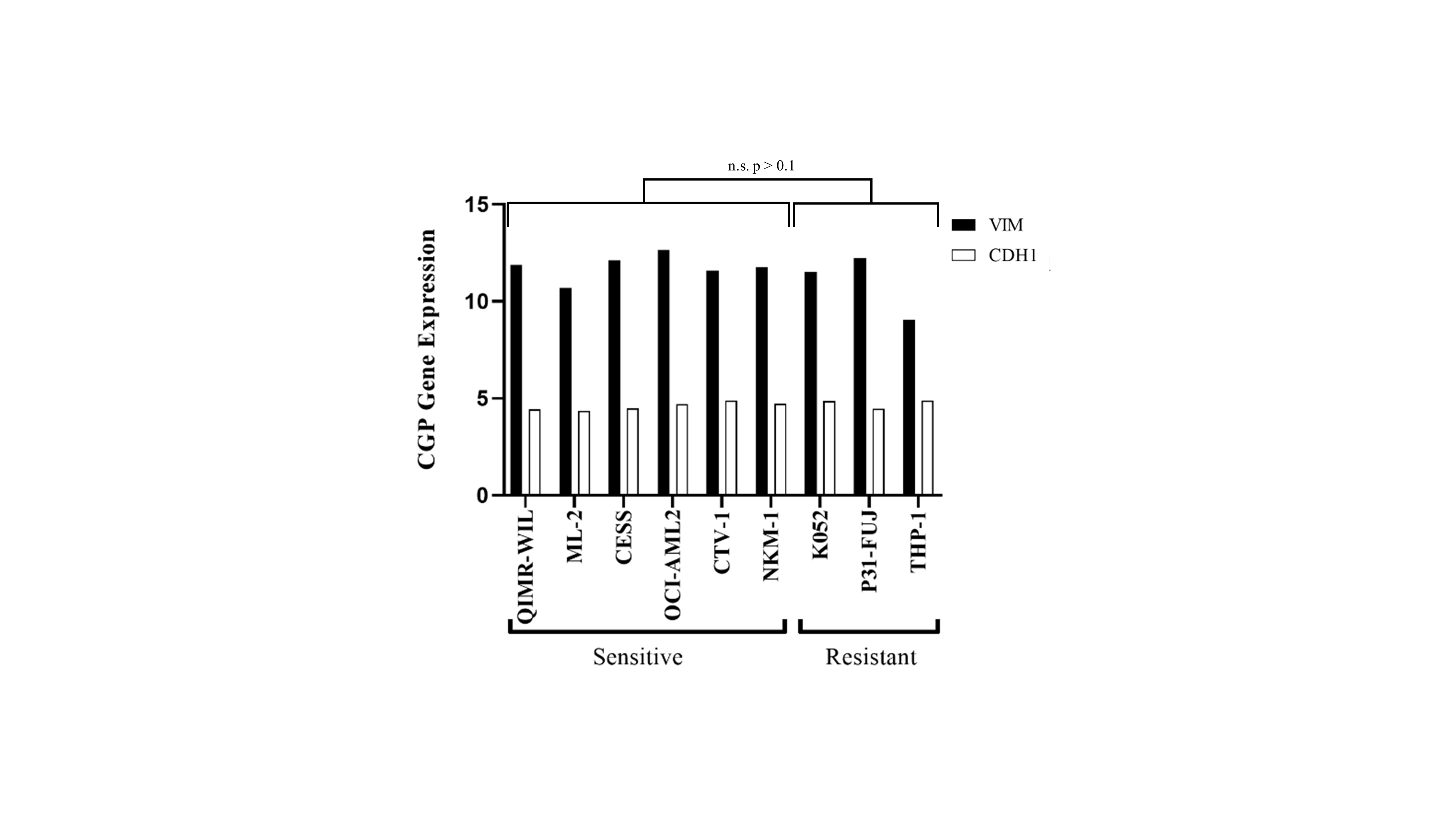

Supplement: S3 Fig — RMA normalized gene expression values of CGP microarray data (y-axis) were used to determine EMT status of sensitive and resistant AML cell lines (x-axis) defined in S1 Fig. VIM: Vimentin (black bars), CDH1: E-cadherin (white bars). n.s. (not significant). (TIF) [file pone.0242497.s003.tif]
