# Supplementary material for: Renin angiotensin system genes are biomarkers for personalized treatment of acute myeloid leukemia with Doxorubicin as well as etoposide
Source: PLoS One. 2020 Nov 25;15(11):e0242497. doi: 10.1371/journal.pone.0242497 (PMC7688131; doi:10.1371/journal.pone.0242497)
Supplement: S2 Table — IC50 values recalculated according to 6M approach using CGP raw cytotoxicity measurements were used to calculate Pearson correlation analysis with CGP IC50 values. Strong correlations are observed for all drugs except for ATRA. (PDF) [file pone.0242497.s005.pdf]

Drugs	r values	p values
ATRA	0.403	0.153117868
Cytarabine	0.984	**6.76E-07**
Etoposide	0.956	**7.93E-04**
Doxorubicin	0.895	**2.84E-01**
