# Supplementary material for: Renin angiotensin system genes are biomarkers for personalized treatment of acute myeloid leukemia with Doxorubicin as well as etoposide
Source: PLoS One. 2020 Nov 25;15(11):e0242497. doi: 10.1371/journal.pone.0242497 (PMC7688131; doi:10.1371/journal.pone.0242497)
Supplement: S3 Table — Eight genes expression data (nine probesets) was used to calculate correlation (as R2 coefficient of determination) with IC50 values of four drugs (ATRA, Cytarabine, Etoposide, Doxorubicin) from CGP database along with recalculated data with 6M approach. Highlighted values with green and red indicate significant correlation in negative and positive manner respectively. (PDF) [file pone.0242497.s006.pdf]

CGP	CTSG 205653_at		CPA3 205624_at		AGT 202834_at		ANPEP 202888_s_at		IGF2R 201393_s_at		RNPEP 208270_s_at		IGF2R 201392_s_at		ATP6AP2 201444_s_at		CTSA 200661_at	
	R^2	P	R^2	P	R^2	P	R^2	P	R^2	P	R^2	P	R^2	P	R^2	P	R^2	P
**ATRA**	0.094	0.248	0.006	0.780	0.052	0.394	0.041	0.450	0.056	0.379	0.054	0.387	0.042	0.445	0.134	0.163	**0.367**	**0.013**
**Cytarabine**	0.106	0.220	0.00004	0.983	0.107	0.216	0.001	0.923	0.017	0.631	0.042	0.448	0.002	0.871	**0.249**	**0.049**	0.128	0.174
**Etoposide**	0.149	0.126	0.048	0.396	0.079	0.273	**0.370**	**0.010**	0.005	0.793	0.001	0.898	0.076	0.286	**0.363**	**0.010**	0.006	0.763
**Doxorubicin**	0.151	0.137	0.067	0.333	0.017	0.629	0.224	0.064	0.075	0.304	0.016	0.636	**0.266**	**0.041**	**0.275**	**0.037**	0.015	0.656

6M IC50	CTSG 205653_at		CPA3 205624_at		AGT 202834_at		ANPEP 202888_s_at		IGF2R 201393_s_at		RNPEP 208270_s_at		IGF2R 201392_s_at		ATP6AP2 201444_s_at		CTSA 200661_at	
	R^2	P	R^2	P	R^2	P	R^2	P	R^2	P	R^2	P	R^2	P	R^2	P	R^2	P
**ATRA**	**0.442**	**0.010**	0.024	0.598	0.060	0.399	0.097	0.280	0.048	0.454	0.092	0.293	0.000001	0.998	**0.362**	**0.023**	0.003	0.843
**Cytarabine**	0.136	0.160	0.007	0.752	0.106	0.220	0.002	0.880	0.008	0.747	0.064	0.344	0.001	0.903	**0.288**	**0.032**	0.138	0.157
**Etoposide**	0.128	0.174	0.124	0.182	0.220	0.067	**0.659**	**0.0001**	0.0005	0.935	0.006	0.768	0.040	0.458	**0.358**	**0.014**	0.101	0.230
**Doxorubicin**	0.210	0.075	0.015	0.649	0.041	0.452	**0.257**	**0.045**	0.015	0.653	0.002	0.870	0.119	0.190	**0.272**	**0.038**	0.085	0.273
