# Supplementary material for: Renin angiotensin system genes are biomarkers for personalized treatment of acute myeloid leukemia with Doxorubicin as well as etoposide
Source: PLoS One. 2020 Nov 25;15(11):e0242497. doi: 10.1371/journal.pone.0242497 (PMC7688131; doi:10.1371/journal.pone.0242497)
Supplement: S4 Table — Adjusted R2 values were calculated in Minitab 17 with eight genes (nine probesets) for four drugs using CGP gene expression data and CGP / 6M IC50 values of the 12 AML cell lines. High correlations are observed with Etoposide and Doxorubicin (bold, for Etoposide R2 > 90%, for Doxorubicin R2 > 80%). Averages of adjusted R2 values of ten randomly divided groups were calculated in Minitab 17 with eight genes (nine probesets) for four drugs using CGP gene expression data and CGP / 6M IC50 values of the 12 AML cell lines. High correlation is observed with Doxorubicin and fine with Etoposide (bold, for Doxorubicin R2 > 85%, for Etoposide R2 > 60%). Asterisk represents the analysis in which Minitab could not perform linear regression analysis. (PDF) [file pone.0242497.s007.pdf]

	AML			
	CGP		6M IC50	
	**R-sq (adj)**	**R-sq (adj) Avg.**	**R-sq (adj)**	**R-sq (adj) Avg.**
ATRA	0.0%	55.45%	*	*
Cytarabine	71.0%	50.32%	73.8%	51.51%
**Etoposide**	**92.9%**	**60.14%**	**98.1%**	**74.39%**
**Doxorubicin**	**81.4%**	**86.14%**	**92.0%**	**94.93%**

* No prospering model could be generated for ATRA by Minitab 17
