# Supplementary material for: Renin angiotensin system genes are biomarkers for personalized treatment of acute myeloid leukemia with Doxorubicin as well as etoposide
Source: PLoS One. 2020 Nov 25;15(11):e0242497. doi: 10.1371/journal.pone.0242497 (PMC7688131; doi:10.1371/journal.pone.0242497)
Supplement: S5 Table — (A) Individual genes and gene combinations were used to generate linear regression models using IC50 values of Doxorubicin and Etoposide from CGP and 6M IC50. Highest correlation is observed in IGF2R/ATP6AP2/CTSA combination with Doxorubicin CGP and 6M IC50 values. And, highest correlation is observed in IGF2R/ATP6AP2/CTSA/CPA3 combination with Etoposide CGP and in ANPEP/ATP6AP2/CTSA/CPA3/AGT combination with Etoposide 6M IC50 values. (B) Regression formulas for gene panels with highest correlations. (PDF) [file pone.0242497.s008.pdf]

A

CGP		
**Doxorubicin**	**R-Sq**	**R-Sq(adj)**
IGF2R	R-Sq = 49.9%	R-Sq(adj) = 37.3%
ATP6AP2	R-Sq = 20.5%	R-Sq(adj) = 11.7%
ANPEP	R-Sq = 15.0%	R-Sq(adj) = 5.6%
CTSG	R-Sq = 6.3%	R-Sq(adj) = 0.0%
CPA3	R-Sq = 3.8%	R-Sq(adj) = 0.0%
CTSA	R-Sq = 2.4%	R-Sq(adj) = 0.0%
AGT	R-Sq = 1.5%	R-Sq(adj) = 0.0%
RNPEP	R-Sq = 0.8%	R-Sq(adj) = 0.0%
IGF2R/ATP6AP2	R-Sq = 68.8%	R-Sq(adj) = 55.5%
***IGF2R/ATP6AP2/CTSA**	**R-Sq = 89.8%**	**R-Sq(adj) = 83.0%**
**Etoposide**		
ATP6AP2	R-Sq = 24.6%	R-Sq(adj) = 17.0%
ANPEP	R-Sq = 20.2%	R-Sq(adj) = 12.2%
IGF2R	R-Sq = 23.6%	R-Sq(adj) = 6.6%
CTSG	R-Sq = 3.0%	R-Sq(adj) = 0.0%
AGT	R-Sq = 1.8%	R-Sq(adj) = 0.0%
CTSA	R-Sq = 1.8%	R-Sq(adj) = 0.0%
RNPEP	R-Sq = 0.4%	R-Sq(adj) = 0.0%
CPA3	R-Sq = 4.2%	R-Sq(adj) = 0.0%
ANPEP/ATP6AP2	R-Sq = 35.8%	R-Sq(adj) = 21.6%
***IGF2R/ATP6AP2/CTSA/CPA3**	**R-Sq = 83.6%**	**R-Sq(adj) = 69.9%**

6M IC50		
**Doxorubicin**	**R-Sq**	**R-Sq(adj)**
IGF2R	R-Sq = 30.1%	R-Sq(adj) = 12.6%
CTSA	R-Sq = 15.6%	R-Sq(adj) = 6.2%
ATP6AP2	R-Sq = 15.1%	R-Sq(adj) = 5.7%
ANPEP	R-Sq = 12.0%	R-Sq(adj) = 2.2%
CTSG	R-Sq = 8.0%	R-Sq(adj) = 0.0%
CPA3	R-Sq = 0.1%	R-Sq(adj) = 0.0%
AGT	R-Sq = 1.2%	R-Sq(adj) = 0.0%
RNPEP	R-Sq = 0.0%	R-Sq(adj) = 0.0%
ANPEP/ATP6AP2	R-Sq = 21.7%	R-Sq(adj) = 2.2%
***IGF2R/ATP6AP2/CTSA**	**R-Sq = 88.8%**	**R-Sq(adj) = 81.4%**
**Etoposide**		
ANPEP	R-Sq = 53.4%	R-Sq(adj) = 48.2%
AGT	R-Sq = 26.1%	R-Sq(adj) = 17.8%
ATP6AP2	R-Sq = 20.4%	R-Sq(adj) = 11.5%
CPA3	R-Sq = 17.1%	R-Sq(adj) = 7.9%
CTSA	R-Sq = 17.1%	R-Sq(adj) = 7.8%
IGF2R	R-Sq = 14.6%	R-Sq(adj) = 0.0%
CTSG	R-Sq = 0.2%	R-Sq(adj) = 0.0%
RNPEP	R-Sq = 3.4%	R-Sq(adj) = 0.0%
ANPEP/ATP6AP2	R-Sq = 60.7%	R-Sq(adj) = 50.9%
***ANPEP/ATP6AP2/CTSA/CPA3/AGT**	**R-Sq = 84.0%**	**R-Sq(adj) = 68.0%**

Selected gene panels

B

**Drug-IC50 origin**	**Selected Gene panels**	**Regression formulas**
**DOXORUBICIN CGP**	***IGF2R/ATP6AP2/ CTSA***	Doxorubicin_IC_50 = 3.66 + 0.710 IGF2R-201393_s_at - 1.29 IGF2R-201392_s_at + 0.510 ATP6AP2-201444_s_at - 0.479 CTSA-200661_at
**DOXORUBICIN 6M IC50**	***IGF2R/ATP6AP2/ CTSA***	Doxorubicin_IC_50 = 2.94 + 0.457 IGF2R-201393_s_at – 0.783 IGF2R-201392_s_at + 0.350 ATP6AP2-201444_s_at – 0.468 CTSA-200661_at
**ETOPOSIDE CGP**	***IGF2R/ATP6AP2/ CTSA/CPA3***	*Etoposide_IC_50 = - 1.08 + 0.517 CPA3-205624_at + 1.40 IGF2R-201393_s_at - 1.24 IGF2R-201392_s_at + 0.724 ATP6AP2-201444_s_at- 1.01 CTSA-200661_at*
**ETOPOSIDE 6M IC50**	***ANPEP/ATP6AP2/ CTSA/CPA3/AGT***	Etoposide_IC_50 = - 0.82 + 0.115 CPA3-205624_at + 0.242 AGT-202834_at + 0.166 ANPEP-202888_s_at + 0.041 ATP6AP2-201444_s_at – 0.258 CTSA-200661_at
