# Supplementary material for: Renin angiotensin system genes are biomarkers for personalized treatment of acute myeloid leukemia with Doxorubicin as well as etoposide
Source: PLoS One. 2020 Nov 25;15(11):e0242497. doi: 10.1371/journal.pone.0242497 (PMC7688131; doi:10.1371/journal.pone.0242497)
Supplement: S6 Table — (A) Expressions of all genes was normalized to GAPDH expression. (B) ATP6AP2, IGF2R (two probesets), CPA3, AGT, and ANPEP gene expression data obtained from CGP in silico and in vitro qRT-PCR expression data from nine cell lines show significant correlations with in vitro qRT-PCR expression data with the exception of CTSA. (PDF) [file pone.0242497.s009.pdf]

A

	ATP6AP2	*IGF2R probe1*	*IGF2R probe2*	*CTSA*	*CPA3*	*AGT*	*ANPEP*
	Relative qPCR Gene Expressions						
**Kasumi-3**	5.62	2.34	2.93	5.05	14.39	12.53	12.70
**GDM-1**	3.62	1.09	0.74	3.75	14.41	5.98	5.17
**CESS**	2.11	1.94	2.39	2.04	7.80	5.64	0.64
**NOMO-1**	5.26	4.95	5.78	5.54	8.02	16.35	7.03
**KASUMI-1**	2.65	2.17	2.58	0.69	3.02	4.58	4.23
**P31FUJ**	4.74	3.24	2.82	3.09	11.17	8.44	9.25
**QIMR-WIL**	3.50	2.81	3.57	2.96	6.02	4.26	0.63
**SKM-1**	4.05	4.39	4.07	3.25	10.32	13.75	8.10
**HEL92.1.7**	2.61	0.25	0.29	0.32	15.77	3.38	8.98

B

**Genes**	**r values**	**p values**
ATP6AP2	0.714	**0.047**
IGF2R probeset1	0.664	**0.073**
IGF2R probeset2	0.705	**0.051**
CTSA	0.065	0.879
CPA3	0.814	**0.014**
AGT	0.908	**0.002**
ANPEP	0.712	**0.048**
