# Supplementary material for: Renin angiotensin system genes are biomarkers for personalized treatment of acute myeloid leukemia with Doxorubicin as well as etoposide
Source: PLoS One. 2020 Nov 25;15(11):e0242497. doi: 10.1371/journal.pone.0242497 (PMC7688131; doi:10.1371/journal.pone.0242497)
Supplement: S7 Table — In vitro IC50 values were obtained from cell viability measurements of the cell lines that are treated with six different concentrations of Doxorubicin and Etoposide separately (20, 10, 2, 1, 0.2, 0.1 μM). Predicted IC50s were calculated using the four formulas generated (with CGP / 6M IC50s) in the linear regression analysis with the normalized gene expression data obtained from qRT-PCR. (PDF) [file pone.0242497.s010.pdf]

	In vitro IC50s		*Predicted IC50s*			
	Etoposide	*Doxorubicin*	*Etoposide CGP*	*Etoposide 6MIC50*	*Doxorubicin CGP*	*Doxorubicin 6M IC50*
**Kasumi-3**	0.251	-0.987	6.523	4.902	3.165	2.048
**GDM-1**	0.550	-0.775	4.877	2.322	2.822	1.932
**CESS**	0.296	-1.139	3.363	1.107	2.956	2.298
**NOMO-1**	-0.056	-0.837	3.228	4.014	1.402	0.952
**KASUMI-1**	0.641	-0.637	2.630	1.271	3.719	3.029
**P31FUJ**	0.552	-0.659	4.931	3.440	2.416	1.902
**QIMR-WIL**	-0.563	-1.447	3.090	0.388	2.937	2.211
**SKM-1**	-0.535	-1.628	4.155	4.367	1.396	1.259
**HEL**	0.135	0.589	8.727	3.326	4.715	3.636
