# Supplementary material for: Renin angiotensin system genes are biomarkers for personalized treatment of acute myeloid leukemia with Doxorubicin as well as etoposide
Source: PLoS One. 2020 Nov 25;15(11):e0242497. doi: 10.1371/journal.pone.0242497 (PMC7688131; doi:10.1371/journal.pone.0242497)
Supplement: S8 Table — (PDF) [file pone.0242497.s011.pdf]

A

NAME	SIZE	ES	NOM p-val	FDR q-val
GO_TUMOR_NECROSIS_FACTOR_RECEPTOR_BINDING	26	0.685	0	0.043
GO_DEFENSE_RESPONSE_TO_VIRUS	125	0.49	0	0.054
GO_RESPONSE_TO_TYPE_I_INTERFERON	62	0.544	0	0.041
GO_LYMPHOCYTE_HOMEOSTASIS	36	0.58	0	0.153
GO_MHC_PROTEIN_COMPLEX	23	0.635	0.002	0.23
GO_INTRINSIC_APOPTOTIC_SIGNALING_PATHWAY_BY_P53_CLASS_MEDIATOR	39	0.553	0	0.193
GO_T_HELPER_1_TYPE_IMMUNE_RESPONSE	18	0.665	0	0.217
GO_ATP_GENERATION_FROM_ADP	35	0.555	0.002	0.193
GO_NUCLEAR_NUCLEOSOME	31	0.582	0	0.205

B

NAME	SIZE	ES	NOM p-val	FDR q-val
GO_REGULATION_OF_TRANSFORMING_GROWTH_FACTOR_BETA_PRODUCTION	25	-0.677	0	0.19
GO_FIBRONECTIN_BINDING	21	-0.693	0	0.164
GO_POSITIVE_REGULATION_OF_FATTY_ACID_BIOSYNTHETIC_PROCESS	15	-0.757	0	0.144
GO_FATTY_ACID_DERIVATIVE_BIOSYNTHETIC_PROCESS	32	-0.617	0	0.134
GO_REGULATION_OF_RENAL_SODIUM_EXCRETION	22	-0.666	0	0.178
GO_REGULATION_OF_RENAL_SYSTEM_PROCESS	35	-0.59	0	0.174
GO_REGULATION_OF_EXCRETION	27	-0.619	0.002	0.152
GO_POSITIVE_REGULATION_OF_COAGULATION	23	-0.638	0	0.154
GO_LEUKOCYTE_CHEMOTAXIS	99	-0.468	0	0.21
GO_REGULATION_OF_COAGULATION	79	-0.488	0	0.196
GO_POSITIVE_REGULATION_OF_FATTY_ACID_METABOLIC_PROCESS	29	-0.606	0	0.206
GO_ATPASE_REGULATOR_ACTIVITY	26	-0.605	0	0.196
GO_TISSUE_REGENERATION	45	-0.545	0	0.207
GO_OXIDOREDUCTASE_ACTIVITY_ACTING_ON_PEROXIDE_AS_ACCEPTOR	33	-0.575	0.002	0.196
GO_MYELOID_LEUKOCYTE_MIGRATION	85	-0.475	0	0.222
GO_METALLOEXOPEPTIDASE_ACTIVITY	36	-0.559	0.002	0.22
GO_DETOXIFICATION	60	-0.502	0	0.214
