# Supplementary material for: Renin angiotensin system genes are biomarkers for personalized treatment of acute myeloid leukemia with Doxorubicin as well as etoposide
Source: PLoS One. 2020 Nov 25;15(11):e0242497. doi: 10.1371/journal.pone.0242497 (PMC7688131; doi:10.1371/journal.pone.0242497)
Supplement: Attachment — Submitted filename: PLOS one revision.docx [file pone.0242497.s014.docx]

Seyhan Turk and co-worker in their work demonstrate that expression of Renin-Angiotensin System (RAS)-related genes predict drug responses (Doxorubicin and Etoposide) in AML patients. Moreover, authors show that identified RAS genes expression stratify AML patients into different subtypes with distinct prognosis. Overall, presented data support use of RAS gene expression analysis as novel tool for AML drug-sensitivity and disease prognostication. Unfortunatley, altough an elegant set of in-silico approaches have been employed, the lack of experimental analyses with appropriate functional in-vitro and in-vivo represents the main drawback of the entire work. In detail:

Major points

•	17 AML cell lines included in CGP database have been chosen for in-silico analysis. In parallel, 9 AML cell lines have been testd for in vitro studies. Are those the same cells included in short list for in-silico analysis? Furthermore, did you see any differences based on their specific genetic background (mutational analysis)?

•	Importantly, GEP analysis have been performed on genes, among those of RAS system, with higher expression variability. Why did you reject those with less variation for your analysis?

•	Could you please detail the NCBI proposed 6-model used approach?

•	To make in vitro date more consostent, could be useful including gene expression analysis as well as IC50 values for all tested AML cell lines.

•	Data showed in Table 2 are not clear. Could you please describe it better?

•	The prognostic role of 3-gene expression signature need deeper analysis. Why did you analyze only 3 genes? What about other RAS genes? Did you perform a cumulative analysis of RAS-related genes?

•	As per Authors own admission, the major study limitation is lack of mutational data analysis. Indeed, it’s worth to be investigated AML patiens subclasses including those carryng poor prognostic mutations such as FLT3. To this aim a detailed description of used AML cell lines could help (i.e. carryng FLT3-ITD or WT, NPM1 etc.)

Minor

- Please pospone figures legend at the end of manuscript right after refernces
- The gene set enrichment analysis revealed findinds that are not supported by experimental data. Overall these data are somehow confusing because are not conclusive at all. In my opinion it’s better including these data as supplementary results to make conclusion more focused
- In the Materials and methods the first sentence of paraghraph is quite misleading. Additionally, please include reference for CGP database.
