# Supplementary material for: Renin angiotensin system genes are biomarkers for personalized treatment of acute myeloid leukemia with Doxorubicin as well as etoposide
Source: PLoS One. 2020 Nov 25;15(11):e0242497. doi: 10.1371/journal.pone.0242497 (PMC7688131; doi:10.1371/journal.pone.0242497)
Supplement: Attachment — Submitted filename: Response to Reviewers.docx [file pone.0242497.s015.docx]

June 20, 2020

Dear Editor,

We would like to thank the Editorial Board and the Referees for all of the important contributions, which will improve the paper.

We have carefully reviewed the comments and revised the manuscript accordingly. Below please find the answers to the Editor’s and Reviewer’s comments.

Yours faithfully,

Dr. Seyhan TURK, PhD

**Journal Requirements:**

**Comment 1.**

1. Please ensure that your manuscript meets PLOS ONE's style requirements, including those for file naming. The PLOS ONE style templates can be found at

https://journals.plos.org/plosone/s/file?id=wjVg/PLOSOne_formatting_sample_main_body.pdf and

https://journals.plos.org/plosone/s/file?id=ba62/PLOSOne_formatting_sample_title_authors_affiliations.pdf

**Response 1.**

PLOS ONE's style requirements fully checked and revisions were done.

**Comment 2.**

2. Please provide additional information about each of the cell lines used in this work, including any quality control testing procedures (authentication, characterization, and mycoplasma testing). For more information, please see http://journals.plos.org/plosone/s/submission-guidelines#loc-cell-lines."

**Response 2.**

Additional information is added for the used cell lines (see *In vitro* section in Materials and Methods).

**Comment 3.**

3. Please provide the source, product number and any lot numbers of the doxorubicin and etoposide obtained for your study.”

**Response 3.**

Drug information has been added to the manuscript.

**Comment 4.**

4. Please note that PLOS does not permit references to “data not shown.” Authors should provide the relevant data within the manuscript, the Supporting Information files, or in a public repository. If the data are not a core part of the research study being presented, we ask that authors remove any references to these data.

**Response 4.**

The “data not shown” was removed and the data was added as “S8 Table”.

**Comments to the Author**

**Reviewer #1:**

**Comment 1.**

The main concern here is about the methodology implied in the first computational section.
I please recommend to specifically indicate, particularly in the method section, if the workflow-analyses performed have been either applied in previous researches or are reported here for the first time. One example is in the “IC50 Calculation Methods” section: the six different models seem introduced by the authors for the first time while, in the result section (line 207) it is referred to them as the “NCBI proposed 6-model approach”, is it the same? Can the author add a reference to this? Finally, I suggest to be more consistent and clearer with the numbers/genes along the text.

**Response 1.**

Here, we report the 6-model (6M) approach for the first time which depends on a non-linear logistic regression function explained in NIH/NCGC assay guidelines. We derived six different versions of this function and select the one with the lowest error rate among all for the calculation of cytotoxicity values. References were added and the “IC50 Calculation Methods” section has been re-written. No inconsistency could be found with the numbers/genes given throughout the entire paper.

**The major points are listed here.**

**Comment 2.**

In the “Data normalization and variance analysis” is there a reason why “the genes whose variance was above 0.8 SD of the mean” were chosen? Additionally, this number is not the same of the results in which is reported “which showed high variation in expression and therefore, selected 9 probesets (8 genes) with standard deviation values above 0.9” (line 205), I would suggest to add a reference or better explain this method. I was wondering why the author did not consider to calculate and consider adjusted p-value for the genes selected.

**Response 2.**

In order to choose the genes which will be used in Real-time PCR for validations, we aimed to choose the most variable genes which could give detectable fold differences in vitro. The variance value of 0.8 was chosen arbitrarily. For these analyses, variance and standard deviation are the same values; we decided to use “variance”. The “Data normalization and variance analysis” section has been expanded and detailed. Since, we are here trying to choose the most variant genes, we did not calculate and consider adjusted p-value for the genes selected.

**Comment 3.**

In “linear regression analyses” section the authors need to better clarify the steps they followed during this methodology, I suggest either to insert some references or clarify the steps. Please also clarify if in this case all the genes or only the 8 were used.

Moreover, I wonder if the Pearson’s correlation was always applied on normal distribution of data, if this is not the case I would suggest a Spearman correlation test.

**Response 3.**

The linear regression analyses section has been re-written. Linear regression analysis was performed with the highly variant eight RAS genes and minimal gene combinations which are now explained more clearly.

We performed Pearson r correlation analysis throughout the paper as we consistently obtained better p values with it, as compared to Spearman’s test.

**Comment 4.**

In the results section the authors refer to 6M data which have not been explained before in the method section, these data likely are deriving from the raw CGP after applying the six model approach, I would suggest to the authors to add this information in the method section.

**Response 4.**

With 6M approach we recalculated IC50 values from raw CGP data for 17 AML cell lines treated with four drugs (ATRA, Cytarabine, Etoposide and Doxorubicin) using an in-house R script. We refer to this data as 6M IC50s.

Additionally, we treated 9 AML cell lines with Doxorubicin and Etoposide *in vitro* and their IC50 values were calculated using 6M approach, as well. We refer to this data as *in vitro* IC50s.

These are explained in the methods section.

**Comment 5.**

I suggest to replicate the random division of the groups and test if the results are consistent with the one obtained here. Moreover, in the method section there is no mention of such a random division, please, add it. If there is a reason why the division was not replicated, please, mention it.

**Response 5.**

In response to this comment we divided 17 cells randomly 10 times and generated 10 different discovery and test groups consisting of 12 cell lines and 5 cell lines, respectively. Linear regression models were generated in the 10 discovery groups separately. The 10 random models of sensitivity to Doxorubicin still have an average R^2^ above 85% for both CGP and 6M IC50, but R^2^ decreased slightly for models of sensitivity to Etoposide. Average R^2^ values now given in “S5 Table”. We added the 10 times random division to the method section and also mentioned it in the results section. However, the reason we performed our analyses without random divisions was because we wanted both the discovery and test cohorts to contain cells that spanned as large a sensitivity interval as possible. We therefore, present in this revised version results from both analyses.

**Comment 6.**

In vitro experiment the genes used are six, and the primers reported in the table 1 are for seven genes. Please clarify this and explain the reason why the authors did not consider all the eight genes from the in silico workflow. Along the text it is not clear if the sub-groups of genes belong to the initial eight. Please reformulate the text in order to give a better explanation of these numbers and other numbers of genes.

**Response 6.**

GAPDH was used as endogenous reference control. That’s why the primers are seven in the Table 1.

We used six genes because in our linear regression analyses, highest correlation is observed in IGF2R/ATP6AP2/CTSA combination with Doxorubicin (both CGP and 6-model) and highest correlation is observed in IGF2R/ATP6AP2/CTSA/CPA3 combination with Etoposide (CGP) and ANPEP/ATP6AP2/CTSA/CPA3/AGT combination with Etoposide (6-model). Prediction model contains totally six genes for Doxorubicin and Etoposide. The section has been re-written for the sake of clarity.

**Comment 7.**

My suggestion is to either reorganize the figures or change the captions: in Fig.3 there is no explanation of the three panels (A, B and C) and neither of the colors. Moreover, beside the Kaplan-Meier curves there are other 3 plots which are not explained. The same for Fig. 4.

**Response 7.**

Figures and their legends were re-organized.

**Minor points:**

**Comment 8.**

- Line 79, when E-MTAB-783 is indicated, please cite the two research articles that contributed to produce these data:

1) Garnett MJ, Edelman EJ, Heidorn SJ, Greenman CD, Dastur A, Lau KW, Greninger P, Thompson IR, Luo X, Soares J, Liu Q. Systematic identification of genomic markers of drug sensitivity in cancer cells. Nature. 2012 Mar;483(7391):570-5.

2) Venkova L, Aliper A, Suntsova M, Kholodenko R, Shepelin D, Borisov N, Malakhova G, Vasilov R, Roumiantsev S, Zhavoronkov A, Buzdin A. Combinatorial high-throughput experimental and bioinformatic approach identifies molecular pathways linked with the sensitivity to anticancer target drugs. Oncotarget. 2015 Sep 29;6(29):27227.

The 1) is already present in the manuscript as ref number 32.

**Response 8.**

Citations were added and the text was reorganized.

**Comment 9.**

-Line 89, please insert the article “the” when referring to the 17 AML and to the 25 genes that are taken by CGP and are indicated in the results section. Moreover, consider to add this info also on the methods.

**Response 9.**

In the method and results sections article "the" were inserted. Source of AML cell lines, CGP, was indicated.

**Comment 10.**
-Lines 121, please insert the website of Minitab 17

**Response 10.**

The website was added.

**Comment 11.**

-Line123-124 if the authors are referring to the same eight genes that have been mentioned in the M&M Normalization section, I would suggest to point it out.

**Response 11.**

These are the same genes, which is now indicated.

**Comment 12.**
-Lines138 Please cite the reference or website for Cluster3.0 and Java Treeview software.

**Response 12.**

The websites for software were added.

**Comment 13.**

- The link at line 144 does not work, please indicate the number of pathways and the number of genes that were present in the C5_all Gene ontology database, and which version of the database was used.

**Response 13.**

The reference was added.

Dataset E-MTAB-783 has 22277 probesets IDs and these were collapsed into 13321 genes. For genes with more than one probeset, one with the highest expression was selected. “C5_all Gene ontology v6.1 database” was used for the analysis which has gene sets that contain genes annotated by the same GO term. Default filtering criteria in GSEA for gene sets is that it should have minimally 15 genes and maximally 500 genes. After applying this filter, analysis was performed for 4081 gene sets. This information was added to the method section, as well.

**Comment 14.**

-Line 188 if the script is available provide it as supplementary information or in a github repository.

**Response 14.**

The R script was provided as supplementary.

**Comment 15.**

-Line 217 the authors are referring to 4 drugs and 8 genes, are these numbers and data the same that have been identified in the previously mentioned analyses? Why did the authors perform linear regression analyses at this step? Please report this information in this section of the manuscript and if the genes/drugs are the ones already mentioned add the definite article “the”.

**Response 15.**

Yes, four drugs and eight genes are the same in the previously mentioned analyses. To examine the correlation of drug sensitivity with combined expression profile of the eight genes, linear regression analyses were performed. “Linear regression analyses” section was reviewed. Article “the” was added when needed.

**Comment 16.**

-Lines 245-247 please refer to which correlation analysis the authors are referring to. Moreover, PLOS does not accept references to “data not shown.”

**Response 16.**

This correlation analysis is referring to test the compatibility of *in silico* and *in vitro* gene expression data with each other. “data not shown” removed from the text and data now given in “S8 Table”.

**Comment 17.**

- Line 248 please indicate why only four formulas were applied and change 4 in four.

**Response 17.**

We applied four formulas since there are four linear regression models for Doxorubicin (CGP and 6M IC50s) and Etoposide (CGP and 6M IC50s) with minimal gene lists, we used these four formulas to predict IC50 values for both drugs in test groups.

“4” was changed to “four”.

**Comment 18.**

-Lines291-292 when the authors refer to “We then stratified patients using the best cut-off values obtained for these 3 genes” please add, “as explained in the method section”.

**Response 18.**

The text was rewritten.

**Comment 19.**
-Line 292 please substitute 3 with three

**Response 19.**

The number was indicated as “three”.

**Comment 20.**

-Figure 1 A) and B) are not indicated. Define the Sy.x parameter, is it the value for the residuals? Please add this information also in the methods.

**Response 20.**

A), B), C) and D) were indicated in the Figure 1.

Sy.x is a standard deviation of the residuals. In our linear regression analyses the residual standard deviation used to describe the difference in standard deviations of CGP and 6M IC50s versus predicted IC50s. It is a goodness-of-fit measure used to show how well our predicted IC50s fit with the CGP and 6M IC50s. It is also mentioned in the methods section.

**Comment 21.**

-Line562 please reformulate “ve resistant”

**Response 21.**

The text was reformulated.

**Comment 22.**
-Uniform the numbers, below 10 the number should be indicated as word.

**Response 22.**

Below 10 the number are indicated as word.

**Reviewer #2:**

**Major points**

**Comment 1.**

• 17 AML cell lines included in CGP database have been chosen for in-silico analysis. In parallel, 9 AML cell lines have been testd for in vitro studies. Are those the same cells included in short list for in-silico analysis? Furthermore, did you see any differences based on their specific genetic background (mutational analysis)?

**Response 1.**

Yes, they are same cell lines. 17 cell lines used in CGP data are listed in S1 Table. These are CTV-1, HL-60, GDM-1, HEL92.1.7, KASUMI-1, KMOE-2, K052, ML-2, MONO-MAC-6, NKM-1, NOMO-1, P31-FUJ, THP-1, QIMR-WIL, CMK, CESS, OCI-AML2.

Cell lines studied *in vitro* experiments are shown in the method section “Cell lines and cytotoxicity experiments”. These are HEL92.1.7, KASUMI-3, GDM-1, QIMR-WIL, CESS, P31/FUJ, NOMO-1, KASUMI-1 and SKM-1.

Seven cell lines used for *in silico* analyses were also used for *in vitro* experiments (HEL92.1.7, KASUMI-1, GDM-1, QIMR-WIL, CESS, P31/FUJ, NOMO-1), as stated in the manuscript.

We performed a comprehensive mutational analysis in order to see if any mutational pattern overlaps with the sensitivity profile, and added the results in the manuscript. Also, nine AML cell lines were WT for NPM1 and FLT mutations.

**Comment 2.**

• Importantly, GEP analysis have been performed on genes, among those of RAS system, with higher expression variability. Why did you reject those with less variation for your analysis?

**Response 2.**

In order to choose the genes that will be used further in Real-time PCR for validations, we first aimed to choose the most variable genes which are also highly likely to give detectable fold differences via PCR.

**Comment 3.**

• Could you please detail the NCBI proposed 6-model used approach?

**Response 3.**

It is detailed in the “IC50 Calculation methods” section.

NCBI methodology or "NIH/NCGC-proposed methodology" suggests a calculation methodology similar to 6-model approach. The function used to model the data is widely being used for cytotoxicity calculations. We derived different versions of this function, which is partly suggested by NIH/NCGC, and select the one with the lowest error rate among all for the calculation of cytotoxicity values.

**Comment 4.**

• To make in vitro date more consistent, could be useful including gene expression analysis as well as IC50 values for all tested AML cell lines.

**Response 4.**

To make more consistent *in vitro* cytotoxicity IC50s, predicted IC50 and QPCR relative gene expression values are now given in “S7 Table” and “S9 Table”.

**Comment 5.**

• Data showed in Table 2 are not clear. Could you please describe it better?

**Response 5.**

The data has been described in more detail.

**Comment 6.**

• The prognostic role of 3-gene expression signature need deeper analysis. Why did you analyze only 3 genes? What about other RAS genes? Did you perform a cumulative analysis of RAS-related genes?

**Response 6.**

First, we started analyzing the 25 RAS genes all together. After applying a variance cut-off, this number decreased to eight genes. After linear regression analysis, these eight genes decreased to six genes (four regression formulas contain totally six genes for Etoposide and Doxorubicin and for two different IC50s (CGP and 6M)). But three genes (IGF2R, CTSA, ATP6AP2) were common for all regression formulas except for one “Etoposide 6M”. Therefore, we analyzed that three genes’ biomarker potential.

Even for Doxorubicin, only these three genes come out in the regression formulas with both CGP and 6M IC50s. Only one additional gene (CPA3) comes out in the regression formula for Etoposide CGP IC50s. Analyses of six genes was necessary to produce predicted IC50s from regression formulas.

**Comment 7.**

• As per Authors own admission, the major study limitation is lack of mutational data analysis. Indeed, it’s worth to be investigated AML patiens subclasses including those carryng poor prognostic mutations such as FLT3. To this aim a detailed description of used AML cell lines could help (i.e. carryng FLT3-ITD or WT, NPM1 etc.)

**Response 7.**

Since there is no mutational data in the patient dataset we could not perform mutational analyses with clinical data. However, we evaluated the mutational profile of 14 out of 17 AML cell lines used in *in silico* analyses. Seven genes (TP53, RBMX, NRAS, ANKRD36C, TNS1, TTN and ASXL1) which are mutated in at least three cell lines were included in mutational analysis and our results are given in “S4 Fig”.

For FLT3 and NPM1 gene, none of the used *in vitro* AML cell lines were mutants.

**Minor points**

**Comment 8.**

• Please pospone figures legend at the end of manuscript right after refernces

**Response 8.**

PLOS Journal requirements state the following: Place figure captions in the manuscript text in read order, immediately following the paragraph where the figure is first cited.

**Comment 9.**

• The gene set enrichment analysis revealed findinds that are not supported by experimental data. Overall these data are somehow confusing because are not conclusive at all. In my opinion it’s better including these data as supplementary results to make conclusion more focused.

**Response 9.**

The gene set enrichment analysis results have been changed as supplementary “S2 Fig” and “S10 Table”.

**Comment 10.**

• In the Materials and methods the first sentence of paraghraph is quite misleading. Additionally, please include reference for CGP database.

**Response 10.**

Paragraph was re-written. The CGP database references were added.
