# Supplementary material for: Renin angiotensin system genes are biomarkers for personalized treatment of acute myeloid leukemia with Doxorubicin as well as etoposide
Source: PLoS One. 2020 Nov 25;15(11):e0242497. doi: 10.1371/journal.pone.0242497 (PMC7688131; doi:10.1371/journal.pone.0242497)
Supplement: Attachment — Submitted filename: Response to Reviewers.docx [file pone.0242497.s016.docx]

**Aug 27, 2020**

**Dear Editor,**

**We would like to thank the Editorial Board and the Reviewer for all the contributions. You can find all the responses and the necessary revisions based on the reviewer’s comments below.**

**Yours faithfully,**

**Dr. Seyhan TURK, PhD**

**Review Comments to the Author**
Reviewer #1: I appreciate the revision of the methods and the extension of the non-linear regression model section, now the methods are sufficiently explained and more clear. However I have noticed that the new version of the manuscript has some parts that need to be reformulated, many typos and inconsistencies along the text.
Additionally it is missing the explanation of the statistics applied in some parts of the manuscript and my concern here is whether or not these tests were rigorously applied.
Finally, I suggest a revision of the Supplementary Materials provided and that the R scripts file will be provided.

For these reasons I am still not considering the manuscript suitable for publication in its current form but I suggest the following points to be addressed to be taken in consideration for publication.

**Response:**

Accordingly, all sections were reformulated. All typos and inconsistencies have been corrected along with the text. All necessary explanations of all applied statistics were added to the manuscript.

All tests were applied rigorously. All Supplementary Materials were reviewed and R script file was provided as a new Supplementary (S2 Table).

**Comment 1:**
Line 82 the authors should insert the references in the correct location, if the reference number 30 is referring to the GSE12417 dataset it should be inserted right after it. As this, there are other similar cases along the text, therefore, I please invite the authors to check this in the manuscript.
**Response 1:**

The locations of all references have been checked and necessary insertions were done.

**Comment 2:**
Line 91, please remove “were chosen arbitrarily”, if any other study used this parameter please cite it.
**Response 2:**

The “were chosen arbitrarily” were removed from the text.

**Comment 3:**
Line 99-100 “explained in De Lean et. al, which is also explained” there is a repetition of the word explain, please substitute with “reported”.

**Response 3:**

The “explained” was substituted with “reported”.

**Comment 4:**
Line129 please, insert here that the IC50 values were included in the CGP. If I am not wrong the authors mentioned this already at line 243.
**Response 4:**

Text was reviewed.

The “IC50 values that were also included in the raw CGP data” and

“We used two versions of CGP data, one original CGP data, second is recalculated 6M IC50 data by R script” were added to the text.

**Comment 5:**
Line 130-131 Please provide the R script and refer to it as supplementary material.
**Response 5:**

The R script was provided in the text and it was referred as a new “S2 Table”

**Comment 6:**

Line 131 “We refer to this data as 6M IC50”, please be consistent with this nomenclature along the text, sometimes it is called “6M IC50” others “IC50 6M” other only “6M”

**Response 6:**

“6M IC50” it was corrected in all necessary locations in the manuscript.

**Comment 7:**

Line 135 “9 AML cell lines were treated with” the number 9 needs to be written in words. Additionally I suggest to reformulate or clarify the meaning of the sentence “IC50 values were calculated using the 6M approach in vitro.”, did the authors mean that the values were calculated using the 6M approach on the data obtained from the in vitro analysis?

**Response 7:**

The number 9 was written in words.

The sentence reformulated as

“In addition, IC50 values were calculated using the 6M IC50 approach on the data obtained from in vitro analysis in which nine AML cell lines were treated with Doxorubicin and Etoposide” to clarify the meaning.

**Comment 8:**

Line 138 8 needs to be written as a word.
The correlations to which the authors are referring here is the one shown in S3 Table, how was this correlation calculated? If it was with graphpad I would suggest inserting a sentence at the end of this section saying that all the correlations were calculated with Graphpad software. Moreover, Pearson correlation should be used when both the variables are normally distributed; in the response to the reviewers the authors mentioned that they got better results with this method but I was wondering if the two variables were tested for normal distribution or not.
**Response 8:**

The number 8 was written in words.

At the end of the section “all the correlations were calculated with Graphpad software” were added.

Pearson's correlation analysis was applied only on normally distributed data. We observed that there was no evidence to reject normality in any variables (p>0.05) except for CPA3 gene (marked in red). The Supplementary data was updated so that an Excel file for the normality test results was added as "Correlation Analyses Results - Kolmogorov Smirnov (KS).

**Comment 9:**

Line 151 please delete “for 10 models” at the end of the sentence, it is redundant.

**Response 9:**

The “for 10 models” was removed from the text.

**Comment 10:**
Line 161 please substitute “that is used to describe” with “that here has been used to describe”.

**Response 10:**

The “that is used to describe” was substituted with “that here has been used to describe”.

**Comment 11:**
Line 173-174 please correct “Gene set enrichment analyses was” with “Gene set enrichment analysis was”

**Response 11:**

The “Gene set enrichment analyses was” was corrected with “Gene set enrichment analysis was”

**Comment 12:**

Line 223 please correct “Clinical data was” with “Clinical data were”

**Response 12:**

The “Clinical data was” was corrected with “Clinical data were”

**Comment 13:**
Line 226 please, when the R script is mentioned in the text, refer to the supplementary material in which it is contained.
**Response 13:**

It was referred to the “S2 Table” when the R script is mentioned in the text.

**Comment 14:**
Lines 228-229 These lines need a reformulation. First, you should put as references the two studies (PMID: 31949498, PMID: 28607584), second, please change “ 'Low' = low expression” with “‘Low’ (low expression)” and “‘High = high expression’” with “‘High’ (high expression)” and finally, substitute “our previous studies” with “as in refX and refY”.
**Response 14:**

The necessary references were added (for PMID: 31949498, PMID: 28607584).

The “'Low' = low expression” was changed to “‘Low’ (low expression)” and “‘High = high expression’” was changed to “‘High’ (high expression)”

And the “our previous studies” was changed to “as in [38,39].”

**Comment 15:**

Line 242 I ask the authors to rewrite the citations when CGP database is mentioned.

**Response 15:**

The citations were rewritten.

**Comment 16:**
Line248 the authors mention: “We observed strong correlations between IC50 values obtained from CGP”, as said above, this correlations need to be clarified in the M&M section.
**Response 16:**

The section were clarified in the M&M section.

The “We performed a Pearson r correlation analysis between CGP IC50s and 6M IC50s to test the compatibility and observed strong correlations between them for drugs widely used in AML (Cytarabine, Etoposide, Doxorubicin) but not for ATRA (S3 Table)” was added.

**Comment 17:**
Line254 The sentence “We then asked whether combined expression analyses of
genes could correlate better with drug sensitivity data.” should be linked with the next paragraph.

**Response 17:**

The sentences were linked together.

**Comment 18:**
Line257-262 please re-arrange these sentences because they are not clear. The explanation of the workflow used has some typos and english grammar errors. Moreover, some parts are already mentioned in the M&M section and should be removed.

**Response 18:**
The section was rearranged.

**Comment 19:**
Line 264 please, remove “RAS genes” it is a redundancy; if it is not, I please ask the authors to explain why it is mentioned here.
**Response 19:**

The “RAS genes” were removed.

**Comment 20:**
TableS4 and S5 should be merged into the same file.
The name of the columns should be revised, precisely: on the top of the column referring to CGP please indicate CGP and on the top of the column referring to 6M IC50 indicate 6M IC50. I also suggest to name the sheets of the .xlsx table according to the table.
The two above mentioned indications are applicable also to the other S Tables.
**Response 20:**

The S4 Table and S5 Table were merged into the same file as a new “S5 Table”.

The name of the columns was revised. CGP and 6M IC50 were indicated on the top of the columns.

The sheets of the .xlsx tables were named according to the tables.

Other S tables were also reviewed.

**Comment 21:**

Line277 6M IC50 needs to be indicated accordingly along the text.

**Response 21:**
“6M IC50” was indicated accordingly along the manuscript.

**Comment 22:**

TableS6, the columns referring to each group need to be marked.

**Response 22:**

S6 Table was revised accordingly.

**Comment 23:**
Figure 1: The names in the title need to be consistent with the content of the text, therefore 6M needs to be substituted with 6M IC50.
**Response 23:**

The names in the titles of the Fig1 were renewed.

**Comment 24:**

Line278 I would suggest to report also here the name of the six genes that have been selected.
**Response 24:**

The gene names were also reported in the text.

**Comment 25:**
Line294 a comma between “ANPEP ATP6AP2” is missing.
**Response 25:**

The comma was added.

**Comment 26:**
I suggest the authors either merge table S7 and S8 or put them in different sheets of the same file.
**Response 26:**

Accordingly, Table S7 and Table S8 were merged as a new “S7 Table”.

**Comment 27:**

S1 Fig: I suggest that the legend of this figure will also include the meaning of the colors. Maybe a scale of colors should be provided in the figure.
**Response 27:**

A color scale was added to the S1 Fig. And the meanings of the colors were added to the legend.

**Comment 28:**

Line320-322 EMT acronym is not explained along the text and there is no mention of the statistical test involved when the authors say: “there were no significant differences between the two groups”. The authors need to mention the test performed (Wilcoxon or t-test according to the distribution of the data) and/or report it in the m&m section.
**Response 28:**

The EMT acronym was explained in the text. The citations were added. We used t-test for statistical test. Accordingly, t-test was mentioned in the text.

**Comment 29:**

S4 Fig needs to be included as a table or a different figure should be provided.
**Response 29:**

S4 Fig was included as a new “S10 Table”.

**Comment 30:**
Line 334-335 I was wondering how the authors investigated the up-regulation of the three genes. Additionally, as previously mentioned from the reviewer 2, it is still not clear to me the choice of these three genes; if it is related to the fact that these genes were the one common for all regression formulas I suggest to mention it in the text and/or m&m section.
**Response 30:**

Accordingly, the sentence was corrected as:

“We found that high expression of genes IGF2R and CTSA were both associated with better overall survival, while the opposite was true for *ATP6AP2* when patients were classified in either “High” or “Low” groups based upon LRMC cutoffs for each gene separately (see Materials and Methods section) (Fig 2).”

The High expression and the opposite were determined using Log-rank with multiple cut-offs (LRMCs) algorithm as described in methods sections under the heading of clinical data validation. LRMC generates all possible cutoffs with their respective p values associated with Hazard ratios (Fig 2A). So for each gene, these cutoffs were generated and one cutoff with the smallest p-value was selected. Patients with expression values above this cutoff were labeled high and the rest were labeled as low. And for these groups, Kaplan Meier graphs were generated (Fig 2B). This explanation is also given in Fig 2 legend along with cutoff values as well.

The reason why these three genes are selected, as explained previously, is because they are common for both Doxorubicin and Etoposide except for Etoposide 6M IC50. As suggested by the reviewer the required explanation has been added to the “Clinical data validation-Log rank with multiple cutoffs (LRMC) and survival analysis” in the M&M section.

**Comment 31:**

Line332 I recommend to add again the reference for GSE12417 when it is mentioned.

**Response 31:**
The reference for GSE12417 was added when needed along with the manuscript.

**Comment 32:**

Line334 Doxorubicin needs to be indicated with the first letter in uppercase.
**Response 32:**

The correction was done.

**Comment 33:**
Line 337 please add in the parenthesis together with Fig2 “see Materials and Methods section”.
**Response 33:**

The “see Materials and Methods section” was added.

**Comment 34:**
Line 339-341 these lines need to be reformulated.

**Response 34:**

The lines were reformulated.

**Comment 35:**

Line352 the word “cut-off” needs to be consistent along the text and the numbers need to be rounded.
**Response 35:**

All “cutoff” terms were made consistent and the numbers were rounded.

**Comment 36:**
Since many S tables are really small I suggest to insert them in a unique pdf file and leave as excel only those that do not fit on a pdf page.
**Response 36:**

Except for S1 Table, all Supplementary Tables were converted to PDF files.

**Comment 37:**
The script file is missing, it should be provided in a .zip file with all the codes.
**Response 37:**

The R Script file has only “one code” and it was provided in the new “S2 Table”.

**Comment 38:**
The section Acknowledgments is blank.

**Response 38:**

Accordingly, the title was deleted because this section is empty.
