# Supplementary material for: Renin angiotensin system genes are biomarkers for personalized treatment of acute myeloid leukemia with Doxorubicin as well as etoposide
Source: PLoS One. 2020 Nov 25;15(11):e0242497. doi: 10.1371/journal.pone.0242497 (PMC7688131; doi:10.1371/journal.pone.0242497)
Supplement: Attachment — Submitted filename: Response to Reviewers.docx [file pone.0242497.s017.docx]

Oct 12, 2020

Dear Editor,

We would like to thank the Editorial board and the Reviewer for all comments. You can find revisions and responses below.

Yours faithfully,

Dr. Seyhan TURK, PhD

**Review Comments to the Author**
Reviewer #1: Turks and colleagues addressed most of the points raised during the last round of revision but one of the most important points, together with some typos/inconsistencies have not been corrected and/or introduced. In order to be accepted I recommend precisely covering all the points raised and to check for possible mistakes newly introduced.

**Main point:** the R script/scripts are still not included in the current version of the manuscript therefore I kindly ask to provide them in one of the following manners: either as file .R / .Rmd in a compressed file (.zip, gzip, tar.gz, ecc ecc) or as a link to a public repository. Currently the only file provided is a 1 x 1 table with the name of the script “SixModelIC50 V3.r” which does not include any code line.

**Response:**

R Scripts were included in the manuscript as a link to a public Github Repository. “S2 Table was removed”

**Minor points to be addressed:**

**Comment 1.**
Line 86-87 It is not clear which dataset has been used for data normalization and variance analysis, the name “CGP microarray” combines the “CGP gene expression data” and the “microarray dataset GSE12417”. I please suggest, if the authors intend the “CGP gene expression data”, to use this name.

**Response 1.**

“CGP microarray” was changed to “CGP gene expression data”.

**Comment 2.**
Line 103 in the new version of the manuscript it comes out that both the models and data have the same name “6M IC50”. This intent was not clear from the previous version, since there was a little bit of confusion in the names. Therefore, I suggest to use two different names for model and data (maybe using lowercase letters in one of the two or only 6M when referring to the model while 6M IC50 when referring to the data). Plase, be consistent along the text with this nomenclature when referring to one or to the other.

**Response 2.**

As suggested by the reviewer, we used “6M” when referring “model” and we used “6M IC50” when referring “data”.

**Comment 3.**

line 131-133: “. We referred to this data as 6M IC50. We used two versions of CGP data, one original CGP data, second is recalculated 6M IC50 data by this script.” Please, reformulate this sentence, it does not seem in the right place and it is not clear. I ask the authors to be consistent with the nomenclature and terms used in the other section.

**Response 3.**

We reformulated and relocated the line.

**Comment 4.**
138-139 the sentence “and observed strong correlations between them for drugs widely used in AML (Cytarabine, Etoposide, Doxorubicin) but not for ATRA (S3 Table).” needs to be moved in the result section. Plus there is a conflict on what it is mentioned in lines 254-256 of the results: “ Using t-test we observed strong correlations between CGP IC50 and 6M IC50, for drugs widely used in AML (Cytarabine, Etoposide, Doxorubicin) but not for ATRA (S3 Table).” The authors need to clarify if they used a t-test or a correlation Pearson test. The table is referring to the Pearson correlation.

**Response 4.**

We removed the “and observed strong correlations between them for drugs widely used in AML (Cytarabine, Etoposide, Doxorubicin) but not for ATRA (S3 Table)” from the Materials and Methods section.

“T-test” was changed to “Pearson correlation” in the Results section.

**Comment 5.**
Line 144: this is the first time the authors refer to the “CGP 6M IC50 data”, please be consistent with the nomenclature as mentioned in the previous paragraph and revision. If the name was only 6M IC50 it needs to be like this, otherwise if a new name is introduced, it needs to be specified before and the authors should explain it.

**Response 5.**

“CGP 6M IC50 data” was changed to “6M IC50 data”.

**Comment 6.**
Line 232 It is quoted another R script that is not the same as the one used to calculate the 6M model but it is referred to as the same. I please ask the authors to correct this, and if it is available, to also provide this script together with the previous one. They can be put together in a compressed (.zip, gzip, tar.gz, ecc ecc) file.

**Comment 6.**

R Scripts were included in the manuscript as a link to a public Github Repository. “S2 Table was removed”

**Comment 7.**
Line 227-228 these new inserted lines need to be reformulated. “IGF2R, CTSA, ATP6AP2 were selected for clinical correlation studies is because they are common for all regression formulas except for Etoposide 6M IC50.” Likely “is because” is a typo, and this is the first time that the authors indicate “Etoposide 6M IC50”, what are they referring to?

**Response 7.**

We reformulated the line.

**Comment 8.**
Line 302 I kindly ask the authors to revise the use of the article “the” in this location. Have these cells been previously indicated in the text of the results? I suggest to remove the “the” and add (see Materials and Methods section) if the authors agree.

**Response 8.**

We removed the “the” and added “(see Materials and Methods section)”.

**Comment 9.**

Lines 324-326 the authors should clarify how they “ tested E-Cadherin (epithelial marker) and Vimentin (mesenchymal marker) expression in silico”, if this analysis is referring to the S3 Fig, I please the authors to add at the end of this paragraph (S3 Fig). Moreover, the following paragraph (Lines 332-334) should not be separated if the authors are agreed. Finally the t-test is not shown in the S3 Fig and therefore the quote “(S3 Fig)” should be removed from line 334.

**Response 9.**

We compared E-Cadherin (epithelial marker) and Vimentin (mesenchymal marker) expression using t-test. We clarified it and showed t-test p value on the S3 Fig.

We added “(S3 Fig)” at the end of paragraph and combined with the next paragraph.

**Comment 10.**

Fig 2 I please ask the authors to explain also the meaning of the red circle in the figure legend. Moreover, I think that the panel A legend needs to be more clear: I find it difficult to read it and it is not explicative of the figure.

**Response 10.**

We simplified the legend, since we already have explanations for this method in methods section and we cited two previous studies. We also explained the meaning of red circle in the legend.
